# Tramadol is a performance-enhancing drug in highly trained cyclists: a randomized controlled trial

**DOI:** 10.1152/japplphysiol.00338.2023

**Published:** 2023-07-06

**Authors:** Alexis R. Mauger, Trudy Thomas, Samuel A. Smith, Christopher R. J. Fennell

**Affiliations:** ^1^School of Sport and Exercise Sciences, University of Kent, Canterbury, United Kingdom; ^2^Medway School of Pharmacy, Universities of Kent and Greenwich, Chatham, United Kingdom

**Keywords:** analgesics, cycling performance, doping, pain, prohibited list

## Abstract

Tramadol is a potent narcotic analgesic reportedly used in multiple sports to reduce exertional pain and confer a performance advantage. This study sought to identify whether tramadol enhances performance in time trial cycling. Twenty-seven highly trained cyclists were screened for tramadol sensitivity and then attended the laboratory across three visits. *Visit 1* identified maximal oxygen uptake, peak power output, and gas exchange threshold through a ramp incremental test. Participants returned to the laboratory on two further occasions to undertake cycling performance tests following the ingestion of either 100 mg of soluble tramadol or a taste-matched placebo control in a double-blind, randomized, and crossover design. In the performance tests, participants completed a 30 min non-exhaustive fixed intensity cycling task at a heavy exercise intensity (272 ± 42 W), immediately followed by a competitive self-paced 25-mile time trial (TT). Following removal of two outlier data sets, analysis was completed on *n* = 25. Participants completed the TT significantly faster (d = 0.54, *P* = 0.012) in the tramadol condition (3758 s ± 232 s) compared with the placebo condition (3808 s ± 248 s) and maintained a significantly higher mean power output (+9 W) throughout the TT (η_p_^2^ = 0.262, *P* = 0.009). Tramadol reduced perception of effort during the fixed intensity trial (*P* = 0.026). The 1.3% faster time in the tramadol condition would be sufficient to change the outcomes of a race and is highly meaningful and pervasive in this cohort of highly trained cyclists. The data from this study suggests that tramadol is a performance-enhancing drug.

**NEW & NOTEWORTHY** In the current study, when cycling with tramadol participants completed a time trial on average 50 s faster and at a 9 W higher power output than the placebo control. The study used both a fixed intensity and self-paced time trial exercise tasks to reflect the demands of a stage race. The outcomes from this study were used by the World Anti-Doping Agency to inform their addition of tramadol to the Prohibited List in 2024.

## Graphical abstract

**Figure:**
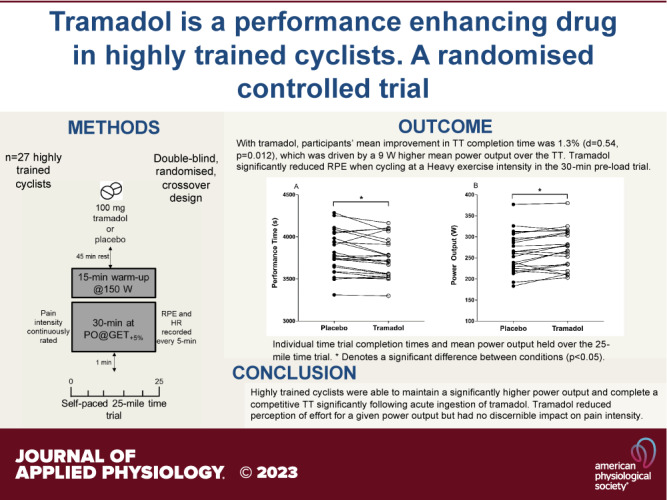

## INTRODUCTION

Tramadol is a synthetic, centrally acting potent opioid analgesic. As a narcotic, tramadol is highly addictive (1), and there are several individual cases where athletes have discussed in media interviews their addiction to opioid use (including tramadol) which has arisen from use in sport. Evidence suggests that tramadol is taken in professional sport where tolerating naturally occurring exertional pain is paramount to success (2–6) and cyclists have previously identified tramadol as a doping agent, inferring riders believe tramadol can be used to enhance performance (4). Thus, even though tramadol presents significant risks to the athlete, the drug has frequently been used not just to treat injury, but to decrease the naturally occurring perceptions of exertional pain and effort that accompany fatigue (7), and therefore gain a performance-enhancing effect.

Although tramadol use has been most prevalent in cycling (showing in 1 in 23 doping controls tested in 2017), the World Anti-Doping Agency (WADA) Monitoring Program (8) found that more than a third of the positive samples for tramadol came from other sports. Therefore, its use and abuse likely go beyond just professional cycling. However, the limited evidence confirming the performance-enhancing effects of tramadol is currently inconclusive. A growing collection of studies (9–11) demonstrate the ergogenic effect of analgesic drugs, yet only three studies examine the effect of tramadol (12–14). The findings of these studies are mixed; however, this is likely due to methodological designs which either do not focus on achieving optimal performance in a physical task (12, 14) or do not account for significant adverse effects of tramadol on individual rider performance in the main analysis (13).

For example, two studies (12, 14) required participants to perform a cognitive task at the same time as the performance time trial to attempt to assess the effects of tramadol on attention. However, these cognitive tasks poorly represent the cognitive/motor control demands of cycling (which might impact physical performance and/or rider safety), and in the participant instructions, it was unclear which task a participant should give priority to or why. When participants who experienced significant adverse effects from tramadol (i.e., vomiting) were removed from the main analysis of the Bejder (13) study, a performance-enhancing effect of tramadol was observed, yet this was not reported in the study’s main conclusions or abstract. All previous studies in this area (12–14) used short performance time trials [20 min (12, 14) or 16 km (13)] which may not represent the types of cycling competition and environment in which tramadol is purportedly taken nor provide an exercise task where management of exertional pain is more likely to improve performance (10). Finally, in previous studies where a pre-fatiguing exercise task (i.e., a “Pre-load” trial) was performed before the time trial (13, 14), this was completed at a power output set according to 60% of peak power output (13) or V̇o_2max_ (14) which was unlikely to induce sufficient pre-load in those participants and could have resulted in participants completing the task in different exercise intensity domains (15).

To address the limitations of the previous literature (12–14), the current study sought to use an experimental design that focused purely on whether tramadol allows highly trained cyclists to maintain a higher power output during a time trial task that more closely reflects the cycling competitions in which tramadol is purportedly taken. Doing this would provide robust experimental evidence to inform whether tramadol should be regulated for in-competition use in sport. Indeed, the data produced from the current study was used by WADA in 2022 to this effect, when it announced its decision to move tramadol to the Prohibited List for 2024 (16).

Therefore, the aim of this study, conducted between 2020 and 2022, was to identify whether acute ingestion of tramadol exerts an ergogenic effect and improves self-paced cycling performance and whether tramadol reduces the perception of pain and/or effort during fixed intensity cycling. It was hypothesized that in comparison to a placebo control, tramadol would significantly improve cycling time trial performance (H1) and would reduce the perception of pain and effort in fixed intensity cycling (H2).

## MATERIALS AND METHODS

### Ethics Approval

This study involved human participants and was approved by the School of Sport and Exercise Sciences Research Ethics Advisory Group (Proposal No.: 36_2019_20) and was conducted in conformity with the Declaration of Helsinki (but without being registered). Participants gave full written informed consent to participate in the study before taking part.

### Participants

Sample size calculations using data from the most comparable study at the time of design (12) showed that an *n* = 27 was required to detect a difference in paired responses at 85% statistical power and 0.05 α. A more recent study with comparable design (13) demonstrated that an *n* = 16 would produce a sensitivity of 7.6 W at a power of 0.8 and α of 0.05.

Participant inclusion criteria were aged 18–55 yr, experience in competing in cycle road racing or triathlon, and the ability to hold a mean power output above 300 W (220 W for females) for a 10-mile TT. Participant characteristics are shown in Table 1. All recruited participants were highly experienced cyclists and were familiar with competing in a range of cycling races.

**Table 1.** Participants’ anthropometric and performance characteristics

Variable	*n* = 27	*n* = 25 (Outliers Removed)
Age, yr	33 ± 10	32 ± 9
Stature, cm	180 ± 7	180 ± 7
Mass, kg	77.9 ± 11.3	78 ± 9.8
Body fat percentage, %	15.4 ± 6.6	15.1 ± 6.3
V̇o_2max_, L/min	4.5 ± 0.5	4.5 ± 0.4
V̇o_2max_, mL/kg/min	58 ± 8	59 ± 8
Peak power output, W	439 ± 56	444 ± 49
Power output at gas exchange threshold + 5%, W	270 ± 44	272 ± 42
Power output at V̇o_2max_, W	410 ± 53	415 ± 48

Values represent mean ± SD for total cohort (*n* = 27 participants) and cohort with outliers removed (*n* = 25).

**Figure 1.**
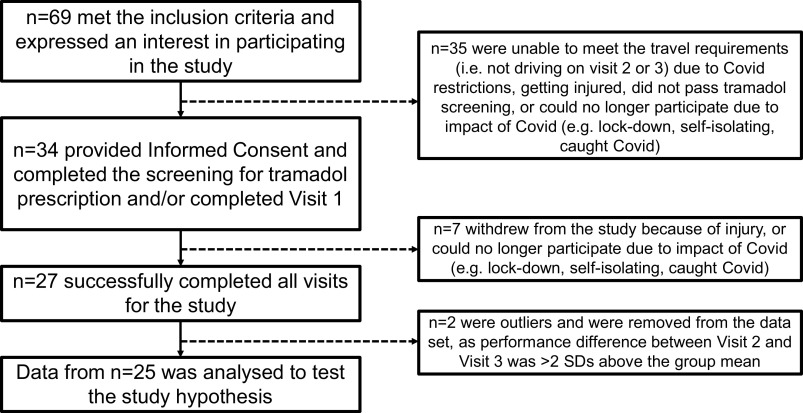
Flow chart detailing participant recruitment and drop-out. The current study started in early March 2020, shortly before the COVID-19 pandemic hit the UK. The UK Government announced the first COVID-19 lock-down on March 23, 2020, and the research laboratories where this study was conducted were closed until October 2020. Two further periods of UK-wide lock-down, and guidance to work from home until February 2022 significantly impacted the recruitment cycles of this project, the retention of participants enrolled in the study, and the length of time the study was conducted over.

Participants were recruited by word-of-mouth, flyers, and social media. For participant recruitment flow chart, see Fig. 1. An *n* = 27 participants completed all experimental procedures.

Before each experimental visit, participants were instructed to avoid vigorous exercise (24 h prior) and abstain from consuming alcohol (48 h abstinence), caffeine (8 h abstinence), and analgesics (12 h abstinence). The study received full ethical approval (Prop 36_2019_20) and was conducted in conformity with the Declaration of Helsinki (but without being registered).

### Equity, Diversity, and Inclusion Statement

Our author team included three men and one woman, two senior and two less-experienced investigators. We stated sex-specific inclusion criteria relating to training/performance status. We offered a £150 time/travel payment to participants to support inclusion. Although our study population included a range of ages within our inclusion criteria, only one female, and two participants from racially minoritized groups participated in the study (see study limitations).

### Study Design

**Figure 2.**
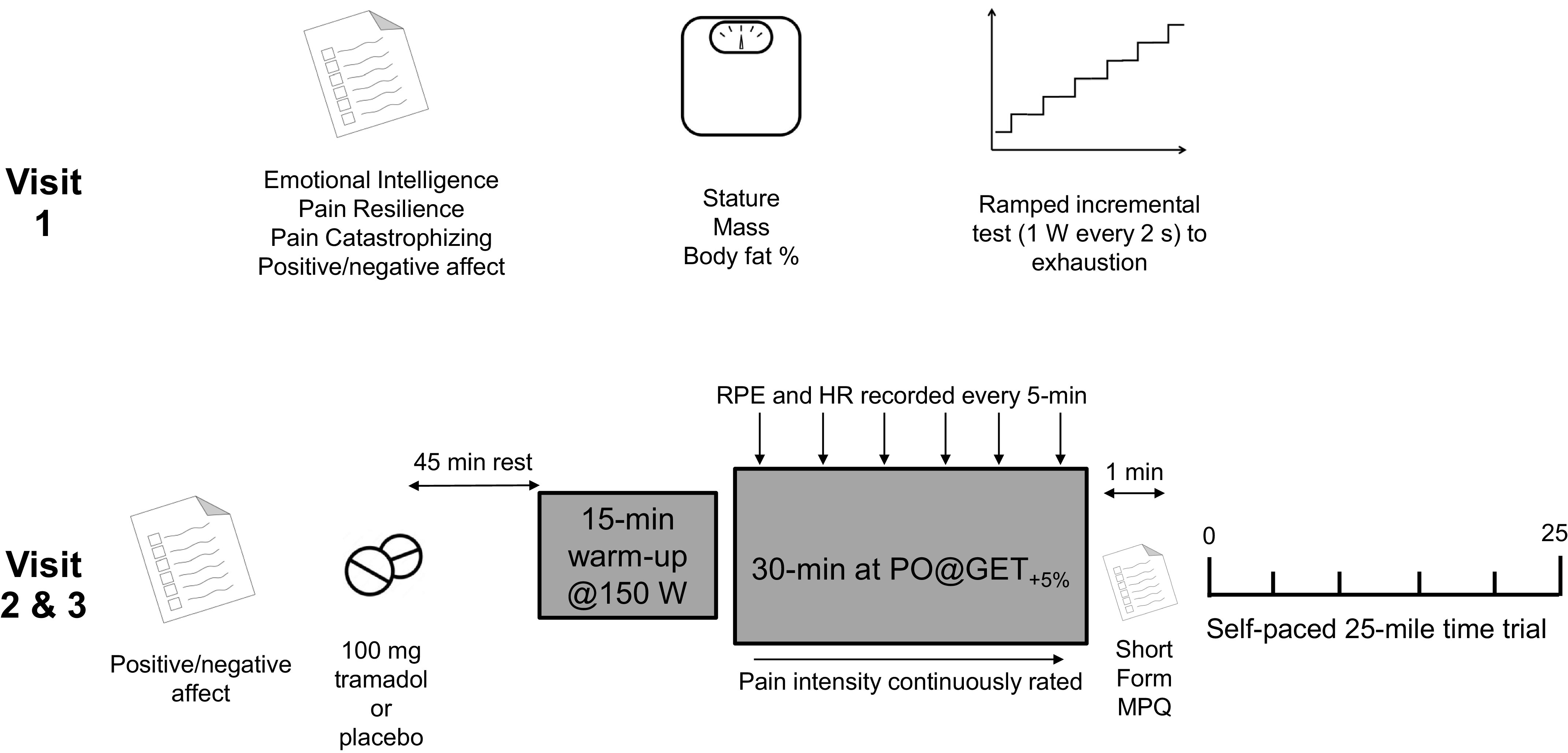
Schematic of the study design and protocol.

This was a randomized, controlled crossover experiment. All participants attended the laboratories at the School of Sport and Exercise Sciences (Kent, UK) on three occasions. The first visit (*Baseline Testing*) identified physiological performance parameters. In two further visits, participants completed cycling performance tests (see *Cycling Performance Testing)* following the ingestion of either tramadol (see *Tramadol Administration)* or a placebo control in a double-blind, randomized, crossover design. Figure 2 shows an overview of the study design.

### Tramadol Screening

Participants were screened for tramadol suitability through a questionnaire and telephone interview with a pharmacist-independent prescriber. On passing this, participants were prescribed the single tramadol dose (see *Tramadol Administration*) and recruited into the full study.

### Baseline Testing

Participants completed a battery of validated questionnaires to identify psychological traits relating to pain experience—positive and negative affect schedule (PANAS) (17), Schutte self-report emotional intelligence test (SSEIT) (18), and the pain resilience scale (PRS) (19). Stature, mass, and body fat percentage (mBCA 525, Seca, Hamburg) were then assessed. Finally, participants completed a ramped incremental test to exhaustion (30 W·min^−1^) on their own race bike (to maximize ecological validity) which was mounted on an electromagnetically braked resistance generator (Cyclus2, RBM elektronik-automation GmbH, Leipzig) to identify maximal oxygen uptake, peak power output, and gas exchange threshold (GET). Gas exchange values determined the “Heavy” exercise intensity for the 30-min non-exhaustive “Pre-load” cycling task on *Visits 2* and *3* (see *Cycling Performance Testing*). Two researchers independently calculated and agreed the intensity at which the GET occurred using the v-slope method (20).

### Cycling Performance Testing

On two further occasions, participants attended the laboratory at the same time of day (±2 h) to complete a 30 min non-exhaustive Pre-load cycling task (Pre-load) followed by a self-paced 25-mile time trial (TT). On entry to the laboratory, participants imbibed their assigned dose of tramadol or placebo (see *Tramadol Administration*) and were asked to sit quietly for 45 min to allow for time to effect. This wash-in period was selected so that peak plasma concentrations of tramadol would coincide with the start of the TT and remain close to peak across it (21–22), with an analgesic effect still likely to be experienced from the start of the pre-load trial (22). Following this, participants completed a 15 min warm-up at 150 W on their own race bike mounted on the same electromagnetically braked resistance generator as Visit 1 (Cyclus2, RBM elektronik-automation GmbH, Leipzig) before commencing the 30 min Pre-load trial which required participants to cycle at a fixed intensity in the Heavy intensity domain (calculated as power output at GET plus 5%; 272 ± 42 W). During the Pre-load, participants verbally reported their rating of perceived effort (RPE) (23) (defined as effort to drive the limb combined with heaviness of breathing) (24) every 5 min, and continuously self-reported their perceived pain intensity on an electronic visual analog scale (25, 26). Participants were instructed to anchor pain intensity according to the worst exertional pain they had previously experienced. One minute after completion of the Pre-load, participants completed a 25-mile (40 km) self-paced TT in the fastest possible time on the same cycle ergometer. During the TT, participants were able to change gearing and cadence and could see the distance they had completed, but they were blinded to all other performance/physiological data (e.g., power output, HR). As a performance incentive, the best performing (fastest mean of TT time in *Visits 2* and *3*) three male and female participants were awarded a “race purse” of £300, £200, and £100 (for first, second, and third place, respectively).

### Tramadol Administration

The tramadol (as Zydol fast-acting soluble 2 × 50 mg tablets) was dispensed by the pharmacy department at the Medway Maritime Hospital. An unblinded investigator dissolved the dose in an opaque water bottle with 100 mL water, before passing this to the researchers administering the test protocol. This dose has previously been shown to induce an effect on µ-opioid receptors, is well-tolerated (21), and broadly elicits an analgesic effect akin to 10 mg of morphine or 6.6 mg of oxycodone (27). The taste and consistency matched placebo was 100 mL water with aniseed/peppermint flavoring and 3 g of inert cellulose powder. As driving is illegal following ingestion of tramadol, *Visits 2* and *3* required participants to make appropriate arrangements to travel home safely.

### Primary Variables

The primary dependent variable was the completion time (seconds) of the 25-mile TT (testing hypothesis 1). The secondary dependent variable was the perceived pain (visual analog scale) and RPE in the 30 min Pre-load (hypothesis 2).

### Statistical Analysis

Differences in TT completion time (hypothesis 1) were tested using a two-tailed paired-samples *t* test. Differences in power output and heart rate during the TT between conditions were assessed using a two-way ANOVA with treatment factor with two fixed levels (TRAM, PLAC) and a repeated measures time factor with five elapsed distances (5, 10, 15, 20, 25 miles). A Pearson correlation was performed on the outcomes from the psychological questionnaires against the difference in completion time between the tramadol and placebo conditions.

Differences in RPE, perceived pain intensity (hypothesis 2), and heart rate between conditions during the Pre-load trial were tested using a two-way ANOVA with treatment factor with two fixed levels (TRAM, PLAC) and a repeated measures time factor with three time-points (10 min, 20 min, and 30 min).

Data are presented as means ± SD unless otherwise stated. All data were checked for the assumptions associated with the statistical tests. For all two-way ANOVAs, a Greenhouse–Geisser correction was used where assumptions of sphericity were violated. Cohen’s d (interpreted as 0.2–0.5 small effect, 0.5–0.8 medium effect, ≥ 0.8 large effect) and partial eta squared (η_p_^2^) (interpreted as 0.01 small effect, 0.06 medium effect, 0.14 large effect) values were used to assess effect sizes. All data analysis was performed in IBM SPSS v26.0 (SPSS, IBM, New York).

For two participants, the difference in TT completion time between the tramadol and placebo condition was an outlier in relation to the wider data set (i.e., difference in completion time between the two conditions was greater than 2 standard deviations outside of the mean of the group), and were removed from the analysis. One of the outliers had a faster tramadol time, the other had a faster placebo time. Key study outcomes were not changed by the removal of these data sets. Due to small sections of missing data during the TT, analysis was conducted on the power output data of *n* = 24 and heart rate data of *n* = 18.

## RESULTS

### Performance Time Trial

#### Completion time.

**Figure 3.**
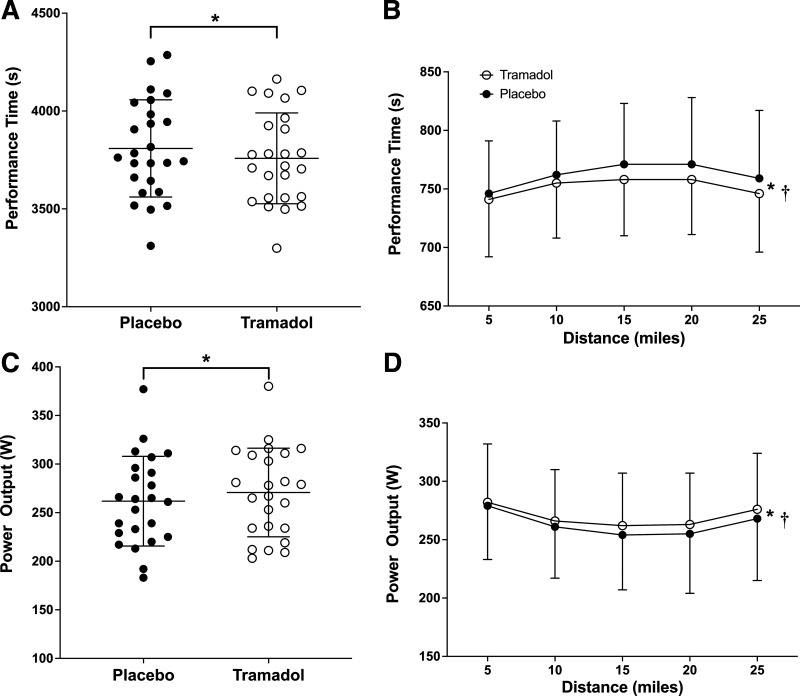
*A:* the 25-mile time trial completion times for participants in the tramadol and placebo conditions. *B*: the participant mean time to complete each 5-mile section of the 25-mile time trial in the tramadol and placebo conditions. *C*: the mean power output that participants rode at in the tramadol and placebo conditions. *D*: the mean power output averaged for each 5-mile section of the 25-mile time trial in the tramadol and placebo conditions. *A* and *C* display the individual performance (circles), the condition mean (center line), and the standard deviation (*top*/*bottom* error bars). *Significant difference between conditions (*P* < 0.05). †Significant main effect of time (*P* < 0.05).

**Figure 4.**
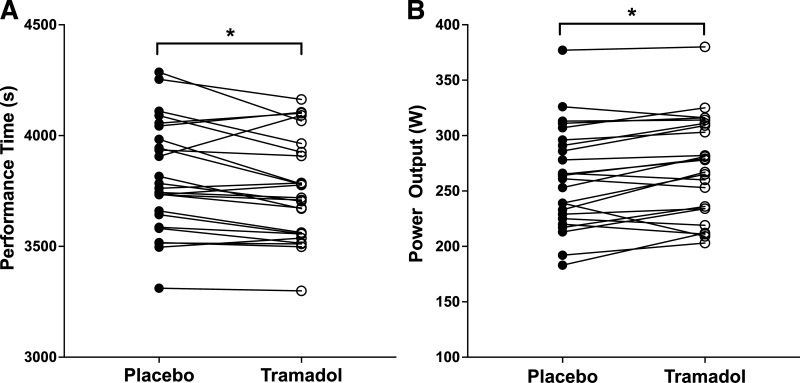
*A*: the 25-mile time trial completion times for individual participants in the tramadol and placebo conditions. *B*: the mean power output each individual participant held over the tramadol and placebo conditions in the 25-mile time trial. *Significant difference between conditions (*P* < 0.05).

Participants cycled the TT significantly faster (*t*_24_ = 2.71, *P* = 0.012, 95%CI_diff_ = 12.11 – 89.23, *d* = 0.54) in the tramadol condition (3758 s ± 232 s) compared with the placebo condition (3808 s ± 248 s). Nineteen of the 25 participants produced faster TT completion times in the tramadol condition, as shown in Fig. 3*A* and 4*A*. For time to complete each 5-mile segment of the TT, there was a main effect of condition (*F*_1,23 _= 7.18, *P* = 0.013, η_p_^2^ = 0.238), and time (*F*_1.54, 35.4 _= 12.37, *P* < 0.001, η_p_^2^ = 0.35), but no interaction effect (*F*_1.77,40.8 _= 1.07, *P* = 0.374, η_p_^2^ = 0.045), as shown in Fig. 3*B*.

#### Power output.

There was a main effect of condition (*F*_1,23_ = 8.17, *P* = 0.009, η_p_^2^ = 0.262), with participants maintaining a higher mean power output during the TT in the tramadol condition (270 W ± 46 W) compared to the placebo condition (261 W ± 46 W), as shown in Fig. 3, *C* and *D*. Individual mean power outputs across the two conditions are shown in Fig. 4*B.* There was also a main effect of time (*F*_1.52, 35.1 _= 14.88, *P* < 0.001, η_p_^2^ = 0.393), but no interaction effect (*F*_1.93,44.5 _= 0.66, *P* = 0.517, η_p_^2^ = 0.028).

#### Heart rate.

There was a main effect of condition (*F*_1,17_ = 6.78, *P* = 0.019, η_p_^2^ = 0.285), with participants maintaining a higher heart rate during the TT in the tramadol condition (171 ± 12 beats/min) compared with the placebo condition (167 ± 12 beats/min). There was also a main effect of time (*F*_1.7,29.2_ = 18.14, *P* < 0.001, η_p_^2^ = 0.516), but no interaction effect (*F*_2.35,39.98 _= 2.13, *P* = 0.124, η_p_^2^ = 0.111).

### Pre-Load Trial

#### Perception of effort.

**Figure 5.**
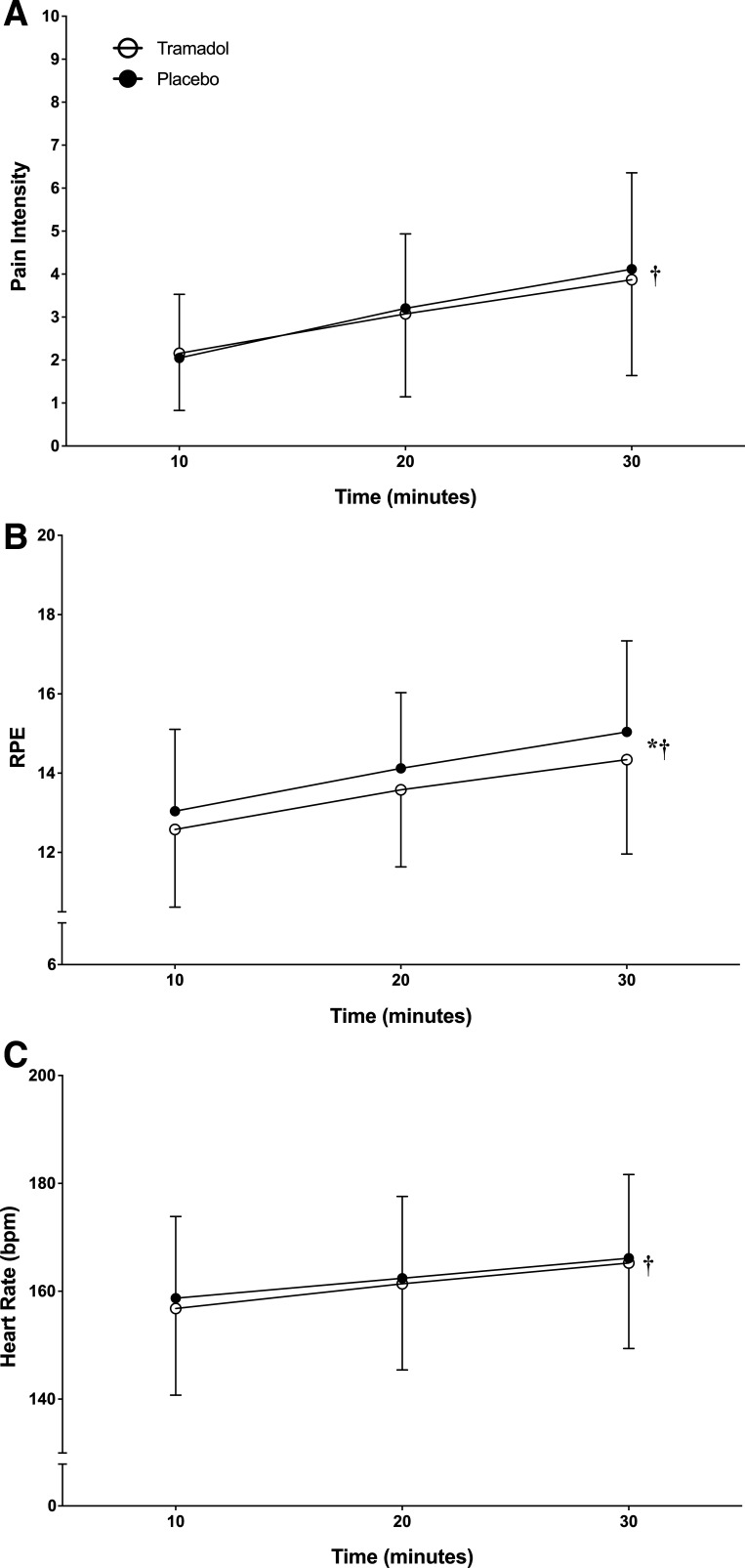
Differences in perceived pain intensity (*A*), perception of effort (*B*), and heart rate (*C*) between conditions in the fixed intensity, 30-min pre-load trial. *Significant main effect of condition (*P* = 0.026). †Significant main effect of time (*P* < 0.05).

There was a significant main effect of condition (*F*_1,24 _= 5.7, *P* = 0.026, η_p_^2^ = 0.191), with participants experiencing a higher mean RPE in the placebo condition (14 ± 0.4 SE) compared with the tramadol condition (13.5 ± 0.4 SE), as shown in Fig. 5*B.* There was also a main effect of time (*F*_1.24,29.7 _= 40.43, *P* < 0.001, η_p_^2^ = 0.628), but no interaction effect observed (*F*_2,48 _= 0.82, *P* = 0.45, η_p_^2^ = 0.033).

#### Pain experience.

There was no main effect of condition for the perceived pain intensity experienced during the Pre-load trial (*F*_1,24 _= 0.24, *P* = 0.63, η_p_^2^ = 0.01). There was a main effect of time (*F*_1.21,29.1 _= 39.2, *P* < 0.001, η_p_^2^ = 0.62), but no interaction effect was observed (*F*_2,48 _= 1.35, *P* = 0.267, η_p_^2^ = 0.054), as shown in Fig. 5*A*.

#### Heart rate.

The heart rate monitor failed to record the data for one participant in the Pre-load trial, so this analysis details *n* = 24. There was no main effect of condition (*F*_1,23 _= 0.98, *P* = 0.33 η_p_^2^ = 0.04). There was a main effect of time (*F*_1.03,23.8 _= 64.2, *P* < 0.001, η_p_^2^ = 0.736), but no interaction effect was observed (*F*_4,46 _= 2.03, *P* = 0.14, η_p_^2^ = 0.08), as shown in Fig. 5*C.*

### Psychological Correlates of Performance

There was a significant correlation between the difference in completion time between conditions and participants’ overall score in the pain resilience scale (*r* = 0.454, *P* = 0.023), with correlations observed in the cognitive/affective positivity score (*r* = 0.503, *P* = 0.01) but not the behavioral perseverance component (*r* = 0.166, *P* = 0.42). No correlations were observed for the PANAS or Schutte self-report emotional intelligence test (all *P* values >0.05).

### Positive and Negative Affect Schedule

All participants arrived in a similar psychological state, with no differences in PANAS results between *Visit 2* and *Visit 3* (all *P* values >0.05).

### Participant Adverse Effects

On completion of the TT, three participants expressed minor adverse effects in the tramadol condition, which included nausea (*n* = 3), mild dizziness (*n* = 3), drowsiness (*n* = 1), and vomiting (*n* = 1). Of these three participants, one produced a faster TT time in the placebo condition, and two produced a faster TT time in the tramadol condition. Removing the participants (*n* = 2) with the most pronounced adverse effects (i.e., drowsiness and vomiting) did not change the main outcomes of the study.

### Blinding

On imbibing the tramadol/placebo solutions, participants were unable to distinguish any differences in taste or texture. However, on completion of all the experimental procedures, when asked which condition they thought they had completed (i.e., placebo or tramadol), seventeen participants correctly guessed the correct intervention, and eight participants incorrectly guessed which solution they received.

## DISCUSSION

This study demonstrates that highly trained cyclists can maintain a significantly higher power output and complete a competitive TT in a significantly faster time following acute ingestion of 100 mg of fast-acting soluble tramadol. Tramadol reduced the perception of effort for a given power output but had no discernible impact on pain intensity whilst cycling. Consequently, hypothesis 1 (H1) was accepted and hypothesis 2 (H2) was partially accepted. The results from this study suggest that tramadol is a performance-enhancing drug in time trial cycling and raises questions pertaining its fair use in competition.

With tramadol, participants’ mean improvement in TT completion time was 1.3%, which was driven by a 9 W higher mean power output over the TT. For a self-paced time trial in a group of highly trained cyclists, this is a significant ergogenic effect. For context, in this cohort of 25 highly trained cyclists a rider with a 1.3% faster TT could change the medalling positions, or take a rider placed in the middle of the third quintile into the middle of the second quintile.

The majority (19 from 25) of participants produced a faster TT in the tramadol condition, and aspects of the Holgado (12) and Bejder (13) studies support this finding. Indeed, the first experiment of the Holgado (12) study demonstrated an 11 W (5%) higher average power output when cycling with tramadol, whilst a 7 W average higher power output was shown for participants who experienced no tramadol adverse effects in the Bejder study (13). No performance-enhancing effect was shown in experiment 2 of the Holgado study (12), but this is likely due to the dual-task employed (i.e., separate physical and cognitive tasks completed in parallel) with participants instructed that the main goal (of the cognitive task) was to be as ‘accurate as possible’. Whilst the Bejder study (13) concluded that tramadol had no performance-enhancing effect, when three participants who exhibited significant adverse reactions to tramadol (i.e., vomiting) were removed from the analysis, a significantly improved performance was detected in the tramadol condition (297 ± 43 W vs. 290 ± 44 W). In competition, it is questionable whether an athlete would take tramadol knowing they were likely to experience adverse effects sufficient to negatively affect their performance. Conversely, for an athlete that does not experience negative side effects and gains a performance advantage from tramadol, they may seek to take a higher dose (i.e., greater than 100 mg) and/or load tramadol over a sustained time period (e.g., several doses across a day), given that the analgesic effect of tramadol is dose-dependent (22). We selected a relatively low dose of 100 mg for this study, to maximize tolerance in this tramadol-naïve cohort, but this means the 1.3% improvement in performance observed here is potentially the minimum ergogenic effect that could be observed in races.

Three of the participants in the current study expressed and displayed adverse effects in the tramadol condition after the TT completion. For one participant these effects were mild (nausea, mild dizziness), whereas for two these were more pronounced (drowsiness or vomiting). It is worth noting that these side effects did not seem to significantly impair their performance (or the ergogenic effect outweighed the impact of the adverse effect), as two of these participants still produced a faster time in the tramadol condition. This is in contrast to the Bejder study (13), where tramadol only seemed to exert a performance-enhancing effect on participants who did not experience pronounced adverse effects.

In the current study, the pre-load trial served to, *1*) induce fatigue in participants before undertaking the TT, thus better replicating the demands of a longer cycle race, and *2*) identify whether tramadol affected the perceptual response to exercise. The key finding was that tramadol significantly reduced RPE when cycling at a Heavy exercise intensity, and it is well evidenced that interventions that reduce the perception of effort for a given exercise intensity result in improved self-paced and fixed intensity time to exhaustion performance (28). However, given the potent analgesic effect of tramadol, it is surprising that no differences in pain intensity were observed in the current study. This may be a result of the electronic visual analog scale used to record pain intensity being over-reliant on participants autonomously self-reporting small differences in pain. Autonomous self-reporting is a different method to how RPE was recorded and whilst it has been used with success in other studies (25–26), these experimentally induced pain rather than alleviated it. Therefore, it may have been challenging for participants in the current study to detect and then autonomously report the more subtle changes in pain arising from tramadol ingestion.

The correlations between the psychometric tests and the differences in completion time are intriguing. They suggest a relationship between the ergogenic effect of tramadol and participants’ pain resilience score, and specifically their cognitive/affective positivity score. In the current cohort, a participant with a higher self-reported pain resilience, and higher perceived ability to regulate emotions and cognition relating to pain was more likely to obtain an ergogenic effect from tramadol. Although this does not demonstrate causation and cannot explain the relationship, it may be that participants who attributed more importance on the impact of pain on exercise performance received an increased benefit for an intervention that mitigated the pain associated with exercise.

### Policy Implications

Combined with the data on the prevalence of use of tramadol in sport (8) and the risks of addiction with continued tramadol use (1), the data from the current study informed WADA’s decision to include tramadol on the 2024 Prohibited Substance List (16).

### Limitations

Positive action was taken to recruit more female participants for this study; however, only one female participant was recruited. Although Holgado et al. (12) identified no differences in response to tramadol between males and females, and the female participant in the current study demonstrated the typical participant response to tramadol (i.e., an ergogenic effect consistent with the group mean), caution should be taken in applying the findings to a female population. The majority of participants in this study came from a White British ethnic group and given that tramadol metabolism is likely to be different between ethnic groups (29), the ergogenic effect and tolerance associated with the dose in the current study should not be assumed outside of a White British cohort.

### Conclusions

The findings from this study suggest that tramadol elicits a significant performance-enhancing effect in highly trained cyclists, such that it can change the outcomes of a race. Given the evidence of the historical prevalence of use of tramadol in sport with the intention of improving performance, and the risks pertaining its use, this study provides strong evidence to justify its inclusion on the 2024 Prohibited Substance List.

## DATA AVAILABILITY

Data will be made available upon reasonable request.

## GRANTS

This work was funded by the World Anti-Doping Agency Research Grants Program (Grant No. 19C03AM).

## DISCLOSURES

The authors report no conflicts of interest or competing interests.

## AUTHOR CONTRIBUTIONS

A.R.M. and T.T. conceived and designed research; A.R.M., T.T., S.A.S., and C.R.J.F. performed experiments; A.R.M., S.A.S., and C.R.J.F. analyzed data; A.R.M., T.T., S.A.S., and C.R.J.F. interpreted results of experiments; A.R.M., S.A.S., and C.R.J.F. prepared figures; A.R.M., T.T., S.A.S., and C.R.J.F. drafted manuscript; A.R.M., T.T., S.A.S., and C.R.J.F. edited and revised manuscript; A.R.M., T.T., S.A.S., and C.R.J.F. approved final version of manuscript.
